# Country differences and determinants of yield in programmatic migrant TB screening in four European countries

**DOI:** 10.5588/ijtld.22.0186

**Published:** 2022-10-01

**Authors:** Dee Menezes, Dominik Zenner, Robert Aldridge, Sarah Anderson, Gerard de Vries, Connie Erkens, Valentina Marchese, Alberto Matteeli, Morris Muzyamba, Joanna Nederby-Öhd, Job van Rest, Ineke Spruijt, John Were, Knut Lönnroth, Ibrahim Abubakar, Frank Cobelens

**Affiliations:** 1 Institute of Health Informatics Research, University College London, UK; 2 Wolfson Institute of Population Health, Queen Mary University London, UK; 3 KNCV Tuberculosis Foundation, The Netherlands; 4 UK Health Security Agency, UK; 5 Università degli Studi di Brescia, Italy; 6 National Institute for Public Health and the Environment, Bilthoven, the Netherlands; 7 Karolinska Institutet, Stockholm Sweden; 8 Faculty of Population Health Sciences, University College London, UK; 9 Universitair Medische Centra, Universiteit van Amsterdam, The Netherlands

**Keywords:** Tuberculosis, screening programmes, migrants

## Abstract

**Introduction:**

The WHO End-TB Strategy emphasises early diagnosis and screening of tuberculosis (TB) in high-risk groups, including migrants. We analysed TB yield data from four large migrant TB screening programmes to inform TB policy.

**Methods:**

We pooled routinely collected individual TB screening episode data from Italy, the Netherlands, Sweden, and the UK under the EU Commission E-DETECT.TB grant, described characteristics of the screened population, and analysed TB case yield.

**Results:**

We collected data on 2,302,260 screening episodes among 2,107,016 migrants, mostly among young adults (aged 18-44, 77.8%) from Asia (78%) and Africa (18%). There were 1,658 TB cases detected through screening with substantial yield variation (per 100,000), being 201.1 for Sweden (111.4-362.7), 68.9 (65.4-72.7) for the UK, 83.2 (73.3-94.4) for the Netherlands and 653.6 (445.4-958.2) in Italy. Most TB cases were notified among migrants from Asia (n=1,206, 75/100,000) or Africa (n=370, 76.4/100,000) and among asylum seekers (n=174, 131.5 per 100,000), migrants to the Netherlands (n=101, 61.9/100,000) and settlement visa migrants to the UK (n=590, 120.3/100,000).

**Conclusions:**

We found considerable variation in yield across programmes, types of migrants and country of origin. This variation may be partly explained by differences in migration patterns and programmatic characteristics.

## Introduction

Globally, tuberculosis (TB) represents a significant burden of disease with 10 million new cases and 1.5 million deaths annually^1^. Progress toward sustainable development goals (SDGs) and World Health Organization (WHO) Global End-TB strategy targets^2^ has slowed down, and potentially reversed during the COVID-19 pandemic^3,4^. Even in low-incidence countries, regaining lost ground^3^ and making sustainable progress toward TB elimination will require effective use of all available tools, including TB screening in specific risk groups^5^.

The TB epidemic in low-incidence countries differs from high-burden countries and is usually concentrated in high-risk groups with higher transmission or higher reactivation risks due to underlying illness or medication, socio-economic circumstances, or higher TB risk in their country of origin. Migrants from high-incidence countries can fall into more than one category. There has been a long history of TB screening in recipient countries, often linked to a health security narrative and related to international borders^6^.

Most low-incidence countries maintain a TB screening programme for inbound migrants, fulfilling certain criteria. These programmes vary substantially in their setting, target groups, screening methods, and in implementation, making comparisons challenging^7^. Previous studies reviewed the effectiveness, cost-effectiveness and impact of these programmes at high level ^8,9^, but direct programme comparisons using primary data are scarce.

The European Commission-funded E-DETECT TB project aims to contribute to *early detection and integrated management of tuberculosis in Europe*^10^, and a key element was to establish a multi-country database on screening for latent and active TB in migrants to allow more granular analysis of these programmes. The aim of this study is to describe and compare the active TB screening programmes in four European countries (Italy, the Netherlands, Sweden and the UK). The comparison focuses on the screened population and programmatic factors to improve understanding of determinants and differences of yield for active TB to inform public health policy.

## Methods

This cross-sectional study is based on a multi-country database, using pooled individual-level data of four TB screening programmes from four European countries with activity between 2005 and 2018 (table 1)^11^. The data sources, pooling and extensive harmonisation process to ensure data can be analysed across these programmes and are compatible with the European Surveillance System (TESSy) standard from European Centre for Disease Prevention and Control (ECDC) has been previously described ^12,13,14,11^. The information from the database was augmented by information from key stakeholders. The aim of this was to capture programme-level information that provide contextual understanding and facilitate data interpretation. The study was based on anonymised observational data, ethics approval was not required.

We carried out descriptive analysis along demographic, clinical and screening/diagnostic characteristics focussing on TB yield; other data on the screening pathway are presented, insofar available.

The main outcome was diagnosis of active TB. To define the outcome, we used a modified version of the EU TB case definition (annex), allowing stratification into possible, probable and confirmed cases^15^. We applied two key alterations to the case definition: (1) all individuals who had a verified record of TB treatment were reclassified as probable cases, independent of whether symptoms were recorded; (2) individuals with a verified record of a positive mycobacterial culture were reclassified as confirmed cases. We present results as yield (expressed as point prevalence) combined and stratified for probable and confirmed cases.

Although some programmes enrol new migrants from countries into a follow-up programme after initial entry screening, our analysis is limited on these (prevalent) cases. In keeping with the Dutch programme definition, Cases notified within 151 days of entry are classified as prevalent cases.

We used simple cross tabulations and graphics to analyse proportions and 95% binomial confidence intervals for proportions, and the χ^2^ or Fisher exact tests as appropriate and explored how programmes and populations vary in their outcomes and to describe patterns of TB case yield variation. Statistical analysis was carried out with STATA 16.1 (Statacorp, Texas, USA).

## Results

### TB screening programmes

Characteristics of the programmes are summarised in table 1. Screening in Italy, the Netherlands and Sweden is carried out on or shortly after arrival; UK screening is done pre-entry in the country of origin by designated clinics. The Netherlands and the UK screen with symptom questionnaires and CXRs, Italy and Sweden offer CXRs to those with symptoms or with a positive TST or IGRA. In Sweden, screening is offered in primary care, in Italy and the Netherlands, screening is offered to asylum seekers in reception centres shortly after arrival or in dedicated outpatient clinics for newly arrived migrants. In the Netherlands, the screening of regular immigrants is offered through the public health service within 3 months of arrival and in Italy it is additionally offered through hospitals. The programmes in Italy and Sweden are voluntary; the Netherlands and UK programmes are mandatory. Italy and Sweden offer screening mainly to asylum seekers. Country of origin incidence thresholds and programmes therefore significantly differ in their scope and size (table 1). Some programmes had changes in these aspects and algorithms during the observation time.

### Screened population

Across all four screening programmes, records of 2,302,260 screening episodes from 2,107,016 individuals were reported. Excluding duplicates (<180 days apart), 195,244 (9.7%) episodes recorded in the UK programme were different screening episodes of the same individuals. These individuals had a median of two screening episodes (interquartile range, IQR 1-2) and an average time of 452 days between episodes.

**Figure 1:**
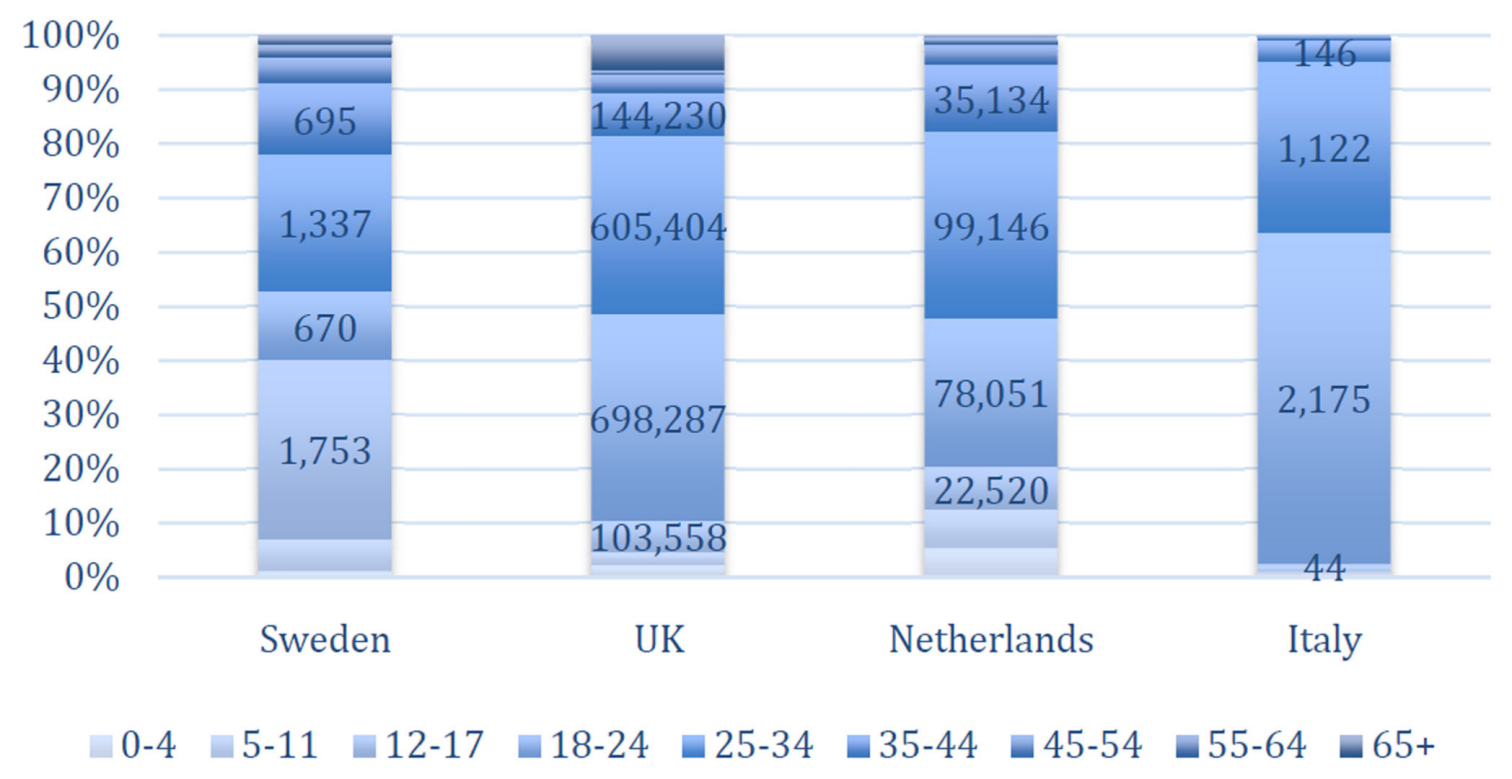
Age distribution of the screened population by screening programme. The numbers on the bars refer to numbers of screens, the vertical axis depicts percentage of age groups among all screens in the respective programme

Most screening episodes were from the UK pre-entry programme (2,006,671, 87.2%) followed by the Netherlands (286,140; 12.4%), Sweden (5,471, 0.2%) and Italy (3,978, 0.2%). Reporting periods varied between programmes and over the years (table 1). Most patients were young adults (aged 18-44, 77.8%), 11.8% were aged 0-17 and 10.4% older than 45 years. Whilst this pattern was similar across programmes, there were notable variations with more children and adolescents in Sweden (40%) and more young adults in Italy (96.6%, figure 1). Slightly more men than women were screened across programmes (male to female ratio 1.11) with significant variations and the ratio ranging between 1.1 (the Netherlands) and 9.8 (Italy).

The migrant typology was variable across programmes and largely reflects the type of programme – in Italy and Sweden all records were from asylum seekers, in the Netherlands the population was split between immigrants (57%) and asylum seekers (43%) and in the UK the majority of screening episodes were among persons with student (45.2%) or settlement visas (24.4%), with lower proportions among those on work visas (7.5%), family reunification (4.3%) and working holiday maker visas (2%). Asylum seekers in the UK undergo domestic health checks and are not part of pre-entry screening.

**Figure 2:**
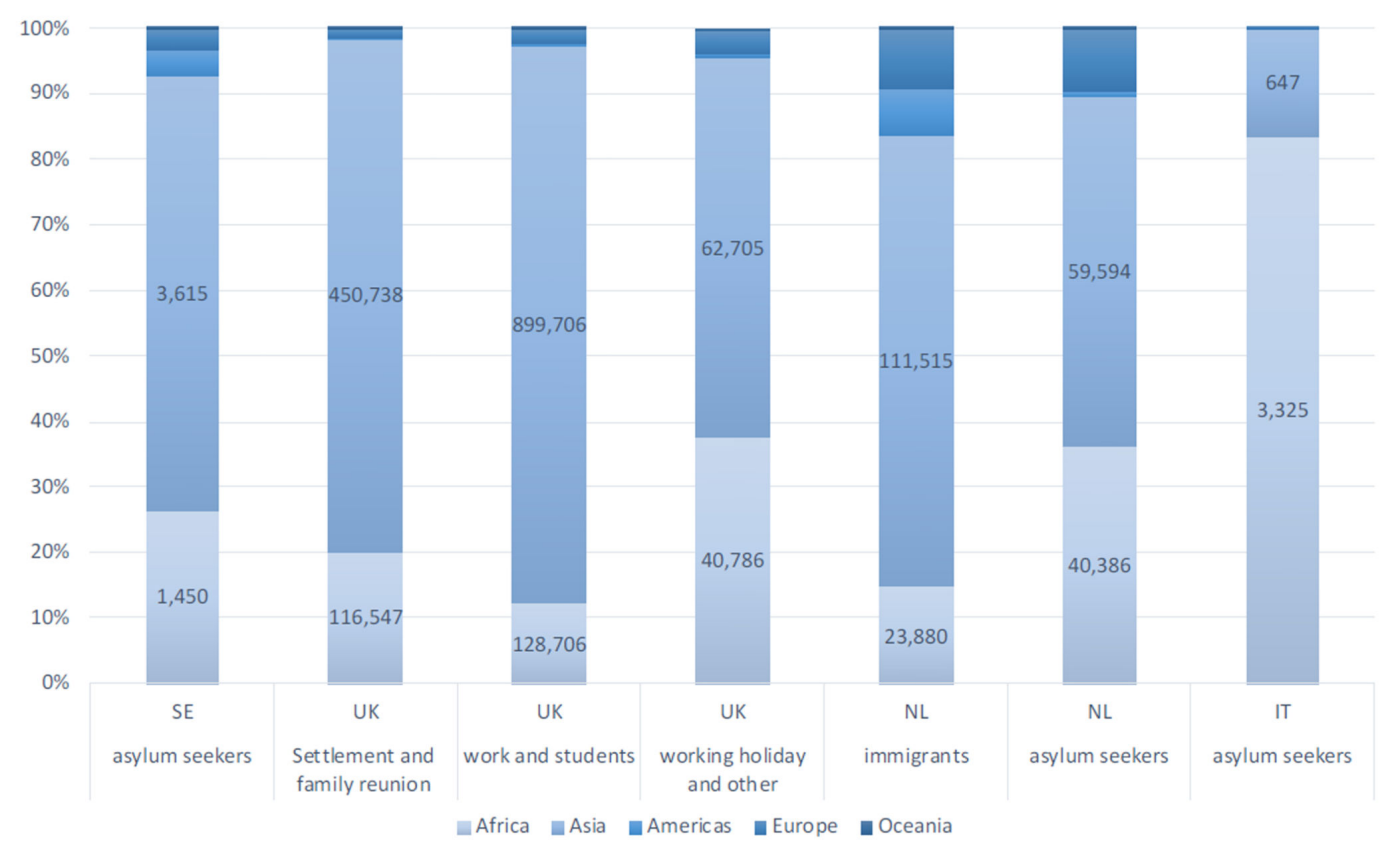
Screened population, by programme, migrant typology and world region of origin. The numbers on the bars refer to numbers of screens (Africa and Asia only), the vertical axis depicts percentage of world regions among the respective migrant type stratified by programme SE: Sweden, NL: the Netherlands, IT: Italy

The most common countries of birth or nationalities were from Asia (78%), particularly from South (46.8%), East Asia (18.7%) and Africa (18%) with smaller proportions from other regions, including Europe (3%), mostly Eastern Europe (2.5%, figure 2). The pattern of distribution across regions was similar across programmes in Sweden, the Netherlands, and the UK, but in Italy there were significantly more migrants from Africa (83.6%) and fewer from Asia (16.3%).

### Active TB

Across the four programmes and all years, there were 1,658 cases (1,278 confirmed and 380 probable) recorded during 2,302,260 screening episodes in total. The crude TB point prevalence rate (yield) was 72.0 (95% confidence interval (CI) 68.6-75.6) per 100,000 persons screened. Most cases were classified as confirmed, both across all (1,278, 77.0%) and in each of the programmes (Sweden 7, 63.6%; UK 1093, 79.0%; the Netherlands 160, 67.2% and Italy 18, 69.2%). For the remainder of the analysis, confirmed and probable cases are analysed together.

**Figure 3:**
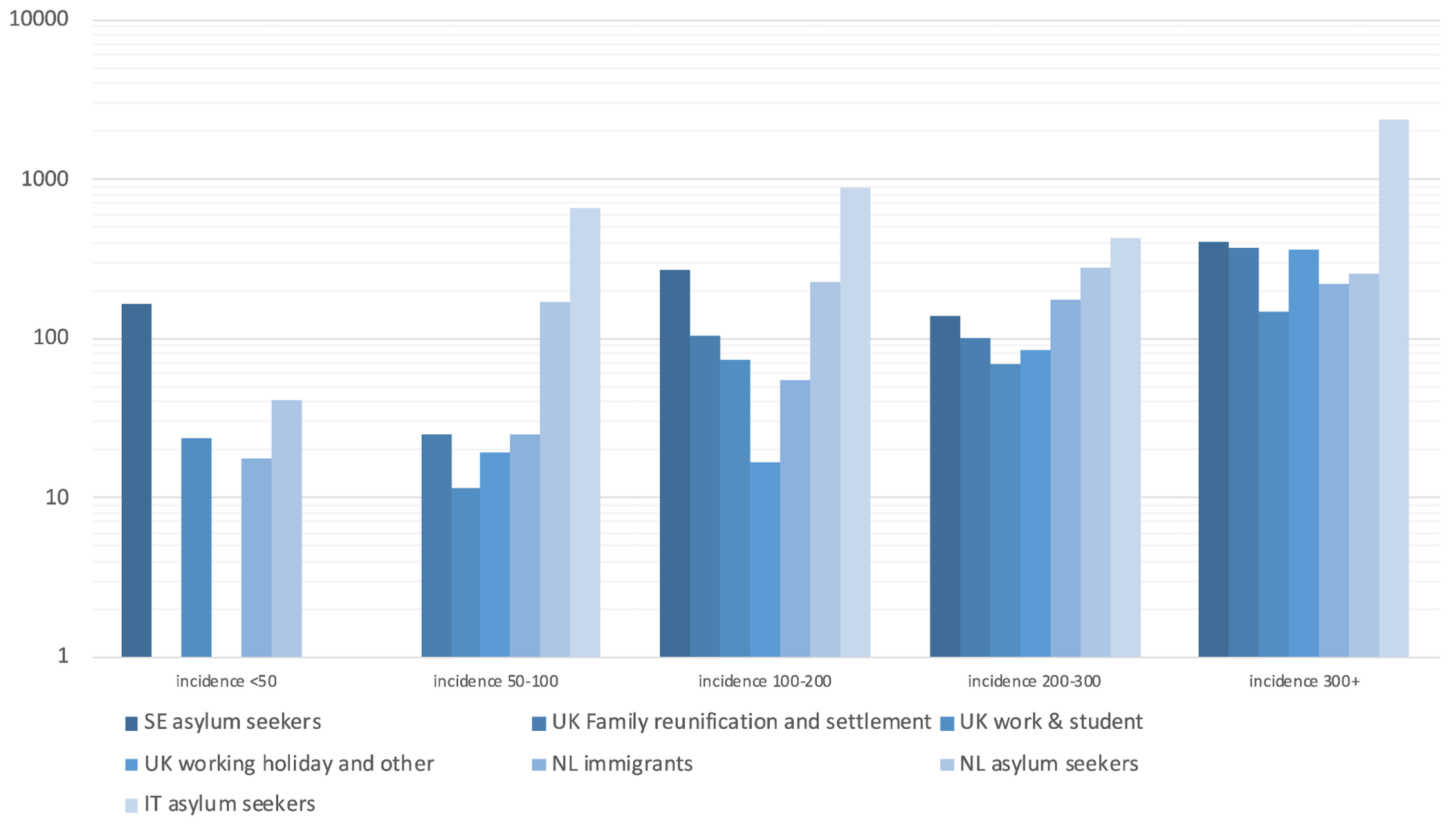
TB yields (rates per 100,000) by programme, countries of birth/ nationalities and migration type. NB: the y axis denotes a logarithmic scale.

The yield per 100,000 varied substantially between programmes, being 201.1 for Sweden (111.4-362.7), 68.9 (65.4-72.7) for the UK, 83.2 (73.3-94.4) for the Netherlands and 653.6 (445.4-958.2) in Italy (table 2). Most TB cases came from migrants with a nationality or country of birth in Asia (n=1,206, 75/100,000) or Africa (n=370, 76.4/100,000) with only a few cases from other regions. In three programmes this distribution was similar; in the Italian programme most TB cases came from Africa (n=25, 751.9 per 100,000 figure 3). The highest three proportions of countries of birth/nationalities recorded among cases differed considerably by programme (table 2).

Of the 2,108,969 episodes with reported CXRs, 2,003,443 (95%) CXRs were reported as normal, 41,776 (2%) as TB-related abnormality, 4,164 (0.2%) as non-TB related abnormality and 59,586 (2.8%) as unspecific abnormality (table 2).

Overall, 8.7 % (n=111) of TB cases had first-line resistances (mostly isoniazid, n=79, 6.2%), including 22 (1.7%) with multidrug-resistant (MDR) TB. This gives an overall estimated prevalence rate of 5.9 and 1.26 per 100,000 for first-line resistance and MDR-TB respectively. No cases of extensively drug-resistant TB were reported. The number and proportion of cultures with first-line resistance and MDR-TB was 0 for both in Sweden, 93 (8.5%) and 12 (1.1%) for the UK, 17 (10.6%) and 9 (5.6%) for the Netherlands and 1 (5.6%) for both for Italy respectively (tables 1,2). Microscopy data was available for 1,398 cases in total and 927 (66.3%) were smear-positive.

Overall, the site of disease for 1,585 (95.6%) of TB cases was pulmonary TB; with a further 37 (2.2%) extrapulmonary, 6 (0.4%) disseminated, 3 (0.2%) lymphatic and 27 other or unknown site (1.6%). In the UK, 98.5% of reported cases were pulmonary disease, whereas in the Netherlands only 84.9% had pulmonary disease. Italy had a significant proportion (23.1%) of disseminated TB.

Overall, a high number and rate of TB cases was recorded among asylum seekers (n=174, 131.5 per 100,000), and high rates and numbers were also reported among migrants to the Netherlands (n=101, 61.9/100,000) and settlement visa migrants to the UK (n=590, 120.3/100,000). A high number but low yield of TB was recorded among UK students (461, 50.8/100,000). UK migrant workers also had an intermediate risk, but lower count (n=111, 74.1/100,000). All other categories had a risk lower than 50 per 100,000 (figure 3).

## Discussion

In our study, we report on a multi-country database containing around 2.3 million TB screening events of migrants to four low-incidence European countries and found similarities and differences in in-bound migration patterns and programmatic differences, including eligibility criteria, migrant population, algorithms, setting and modalities of screening^7^, leading to different yields for active TB. We also observed several programmatic and outcome changes over time. Although some factors had been previously described resulting in recommended targeted approaches ^8,6^, the extent of variation was surprising warranting further investigation.

A number of previous studies have investigated factors associated with yield, including setting^8,17,18^, the relevance of incidence threshold levels^9,19,20^ or migrant typology^12,14^, but few quantified how these factors play out in relation to each other in different programmes and countries. Whilst these factors^9^ apply to all programmes, major programmatic factors may help additionally explain yield variations. The algorithms, including the combination and sequence of tests differ, and the combination of tests or the pre-selection of cohorts by test can have an effect on yield. The logic of high-sensitivity initial testing, followed by high-specificity testing is common in other screening programmes^21^, but has not led to harmonised practice throughout Europe^7,22^ and specific policy preferences can lead to offering screening to lower risk migrants (e.g. students)^14^.

The observed variation in yield is additionally explained by the way the screening programmes are organised. In Sweden, TB screening is offered on a voluntary basis to all asylum seekers and specific other categories of migrants (refugees and family reunification visas). It always includes ruling-out active TB by symptom-check and can include LTBI screening and CXR for those with symptoms or positive LTBI test^13^. The Italian programme shows several important characteristics, which in combination could explain the higher screening yields, for example a more targeted screening approach, compared with the broader UK programme. Similar to the Swedish programme, the Italian programme is also integrated with LTBI screening, offered on a voluntary basis mainly to asylum seekers and the algorithm includes CXRs for those with symptoms or positive LTBI test^23^. Selecting populations for CXR screening based on a (pre-)test, such as a symptom or IGRA screen, could result in similar overall TB yields with less CXRs done, but may miss pre-test negative cases.

These programme-level variations are often contextual and not always undesirable. For example, Italy’s focus on screening asylum seekers who have recently arrived in Europe results in a screened population with a high background incidence rate (from Sub-Saharan Africa) and possibly higher recent TB risks *en route.* Italy’s geographic location makes it an important receiving country of irregular arrivals from Libya by boat during the period examined here and first arrival centre for migrants (including those with onward travel). The higher TB risk among persons from specific African countries has also been described in other destination countries^24^, albeit less dramatically. Hazards along the Central Mediterranean Route are well described^25^ and may explain findings of higher TB incidence among specific migrant typologies, such as asylum seekers or refugees^26^. Setting and population specificity should be a key consideration, when designing effective TB screening programmes for migrants.

Our study benefits from pooling four large, relatively complete programme datasets making a comparison of individual outcomes between these programmes possible. Notwithstanding extensive cleaning and harmonisation, merging observational datasets designed to allow monitoring of screening programmes leads to important limitations, related to data entry, including missing data and potential for misclassification. The distribution of missing data is variable and can be high for some exposure factors (annex). It is possible that missing data or misclassification led to under-ascertainment, although the primary outcome and key exposure variables had a high level of completion.

The data harmonisation between countries presented important challenges, caused by different classification standards. Some variables had to be reclassified to allow harmonisation of datasets, for example country of birth was replaced with nationality, if the former was not available and age could only be analysed as categorical variable, since age only provided as such by some programmes.

Finally, our findings are not generalisable to all migrants, they are representative within the context of these screening programmes. For example, the programs in the UK, the Netherlands and Sweden only screened those whose country of origin had an WHO-estimated incidence above a certain threshold and some countries were exempt from screening by virtue of international regulations (e.g., within EU). Programmes and screening population may change over time, often informed by evaluations^12^ and attempts to generalise our findings need to be mindful of such changes.

In conclusion, we explored programme- and individual-level variations regarding TB screening yield in four important European migrant screening programmes. We found significant variability of these programmes in location and time, leading to highly variable outcomes only partly explained by the demographics of the screened population.

Variation in screening is a result of historical and contextual developments. Nevertheless, it is important to identify best practice and to understand variation and inform guidance based on that, with remaining expected variation minimised. Our study is a first step in this process, informing policy and data collection together with ECDC and WHO and our data may form the basis for a European data collection system with the aim of informing homogeneous policies.

## Figures and Tables

**Table 1:** Basic characteristics of the four included screening programmes. Repeated culture denotes cultures on different specimens and days.

	**Italy**	**Netherlands**	**Sweden**	**UK**
**Setting**	asylum centres	on entry/ reception centre, follow-up in community	primary health care	pre-entry
**Target population**	asylum seekers, new arrivals	New migrants and asylum seekers from non-EU countries with TB rate >50 per 100,000 (before 2015 all immigrants and before 2016 all asylum seekers) with intention of stay >3 months	asylum seekers and refugees are actively invited. Others (new arrivals from non-EU countries with TB rate >100 per 100,000 within two years are eligible	visa applicants from countries with TB rate> 40 per 100,000 if intending to stay 6 months or more
**Mandatory?**	No	Yes	No	yes
Screening tests	IGRA/TST +symptom check/ CXR	symptom check/ CXR	TST/IGRA, symptom check/ CXR if any positive	symptom check/ CXR
**Diagnostic tests**	culture/ molecular tests	Smear /culture/ molecular tests	culture/ molecular tests	smear and 3x culture
**M/F ratio**	9.77	1.1	2.23	1.25
**Time frame**	2015-2018	2011-2017	2015-2018	2005-2018
**Screens per year (mean and SD)**	723 (646)	40,887 (10,648)	1,368 (1,025)	143,226 (93,819)
**Total screening episodes**	3,978	286,140	5,471	2,006,671

IGRA – Interferon Gamma Release Assay, TST: Tuberculin Skin test, M/F ratio: male female ratio.

**Table 2:** Numbers and rates of tuberculosis cases including drug-resistance recorded in four programmes.

	Italy	The Netherlands	Sweden	UK	Total
**Total screens**	3,978	286,140	5,471	2,006,671	2,302,260
**Probable and confirmed TB cases**	26*	238**	11	1,383	1,658
**rates (per 100,000) of probable and confirmed TB cases (95% CI)**	653.6 (445.4-958.2	83.2 (73.3-94.4)	201.1 (111.4-362.7)	68.9 (65.4-72.7)	72.0(68.6-75.6)
**Top 3 countries of birth/ nationalities (numbers and % of prevalent cases)**	Gambia (5, 19.2%) Nigeria (5, 19.2%) Côte d’Ivoire (4, 15.4%)	Eritrea (30, 12.6%) Somalia (22, 9.2%) Indonesia (18, 7.6%)	Afghanistan (5, 45.5%) Congo, DRC, Ethiopia, Iraq, Mongolia and Somalia (each 1, 9.1%)	Pakistan (244, 17.6%) Philippines (216, 15.6%) Thailand (202, 14.6%)	
**TB cases with abnormal CXR (% of all TB cases)**	24 (92.3)	190 (85.6)	8 (80)	1,299 (95.8)	1,521 (94.2)
**Numbers of all culture confirmed TB cases (% of all TB cases)**	18 (69.2)	160 (67.2)	7 (63.6%)	1093 (79.0)	1278 (77.1)
**rates (per 100,000) of culture confirmed TB cases (95% CI)**	527.9 (344.4-808.3)	84.2 (74.2-95.6)	128 (61.0-268.1)	54.5 (51.3 57.8)	59.2 (56.1-62.4)
**MDR (% of culture confirmed)**	1 (4.8%)	14 (5.8%)	0	12 (1.1%)	29 (1.9%)
**any first line resistance (% of culture confirmed)**	1 (4.8%)	28 (11.6%)	0	94 (8.6%)	122 (9.0%)

MDR: Multidrug resistant TB, CXR: chest X-ray, prevalent TB: detected at or <151 days post screening, CI: 95% Confidence intervals. TB yield for all cases includes both “probable” and “confirmed” TB diagnoses.

* Italy had 6 additional incident cases.

** The Netherlands had 139 additional incident cases.
